# Supplementary material for: Anisotropic Superconducting Gap and Elongated Vortices with Caroli-De Gennes-Matricon States in the New Superconductor Ta4Pd3Te16
Source: Sci Rep. 2015 Mar 23;5:9408. doi: 10.1038/srep09408 (PMC4369749; doi:10.1038/srep09408)
Supplement: Supplementary Information [file srep09408-s1.pdf]

Supplementary Information

Anisotropic Superconducting Gap and Elongated Vortices with Caroli-De Gennes-Matricon States in the New Superconductor $Ta_4Pd_3Te_{16}$

Zengyi Du, Delong Fang, Zhenyu Wang, Yufeng Li, Guan Du, Huan Yang*, Xiyu Zhu, & Hai-Hu Wen*

Center for Superconducting Physics and Materials, National Laboratory of Solid State Microstructures and Department of Physics, Collaborative Innovation Center for Advanced Microstructures, Nanjing University, Nanjing 210093, China

I. STS spectra and theoretical fitting with different gap functions

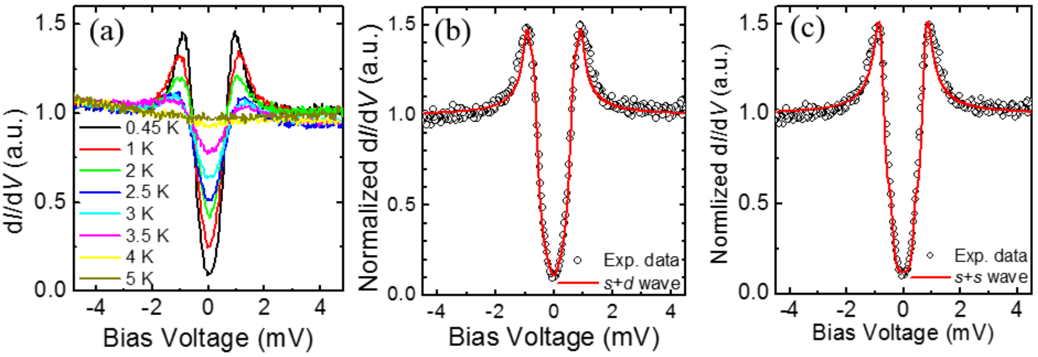

Figure S1 | Temperature dependence of tunnelling spectra and theoretical fitting. (a), Temperature dependence of tunnelling spectra measured from 0.45 K to 5 K. (b,c), Experimental d*I*/d*V* curve and the fitting curve with different gap functions in $Ta_4Pa_3Te_{16}$ single crystal at 0.45 K. The symbols represent the experimental spectra at 0.45 K normalized by the one measured in normal state (at 5 K), the solid line in (b) shows for *d*+*s* wave, that in (c) shows for s_1+s_2 wave fitting based on the Dynes model.

Temperature dependent tunnelling spectra are displayed in Fig. S1(a) from 0.45 K to 5 K (above the critical temperature of 4.5 K). In order to get a more quantitative understanding of the superconducting parameter, we use the Dynes model to fit a typical STS spectrum at 0.45 K normalized by the one measured in the normal state (at 5 K). Dynes noted that the superconducting DOS could be generalized to take into account a finite quasiparticle lifetime by writing

$$N_S(E,\Gamma) = \mathrm{Re}\left(\frac{\varepsilon - i\Gamma}{\sqrt{(\varepsilon - i\Gamma\,)^2 - \Delta^2}} \right) , \tag{S1}$$

So the tunnelling current measured with STS can be written as

$$I(V) = \frac{1}{2\pi}\int_{-\infty}^{+\infty} d\varepsilon [f(\varepsilon) - f(\varepsilon + eV)] \cdot \mathrm{Re}\left(\frac{\varepsilon + eV + i\Gamma}{\sqrt{(\varepsilon + eV + i\Gamma)^2 - \Delta^2}} \right) . \tag{S2}$$

Here $f(\varepsilon)$ is the Fermi function, and Γ is the inverse quasiparticle lifetime. We firstly performed fits to our experimental data using two single-band models, namely $\Delta = \Delta_0$ for an isotropic *s*-wave gap model and $\Delta(\theta) = \Delta_0 |\cos 2\theta|$ for a *d*-wave gap model. The results have been shown in Fig. 2(c). Concerning the structure of the material, a two-fold-symmetric anisotropic s-wave gap function is also used to fit the spectra. In this case, the gap function can be written as $\Delta(\theta) = \Delta_1 + \Delta_2 \cos 2\theta$. The best fit is shown in Fig. 2(d), which leads to $\Delta(\theta) = 0.644 + 0.276 \cos 2\theta$ with the maximum and minimum gaps of 0.92 and 0.37 meV, respectively.

When using a simplified two-gap model to fit the normalized spectra, the combined differential conductivity can be constructed as $G = p dI_1 / dV + (1 - p) dI_2 / dV$, where $I_{1(2)}(V)$ is the tunnelling current contributed by the two gap $\Delta_{1(2)}$ and *p* is the related spectral weight of each band contributed to the tunnelling current. Each $I_{1(2)}(V)$ can be

described by equation S2. The fitting curve obtained by using two-band model (s_1+s_2 or s+d, each band with 50% weight) has been displayed in Fig. S2(b). In the case of d+s, the theoretical curve can also describe the experimental data quite well.

II. Treatment of vortex lattice with Fourier transform analysis procedure

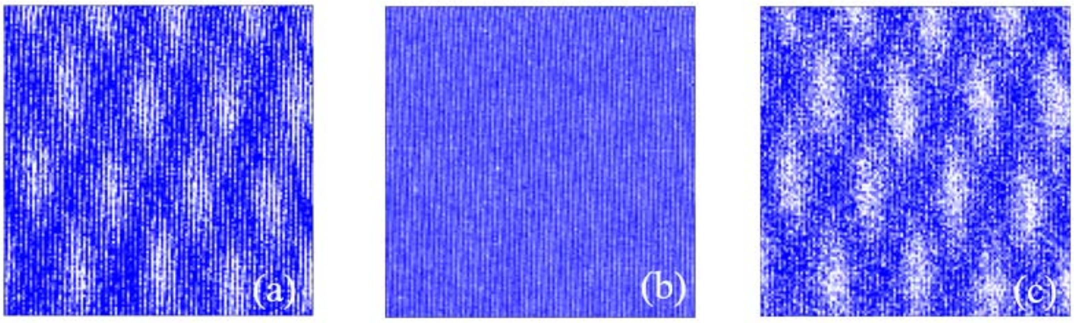

Figure S2 | Vortex image measured at 0.45 K and 0.8 T after the treatment. (a,b), The raw data of differential conductance image $g(\vec{r},E)$ of E = 0 and E = 0.95 meV in an area of 180×180 nm^2, respectively. (c), The difference of the differential conductance image by taking $g(\vec{r},E=0\ \text{meV})-g(\vec{r},E=0.95\ \text{meV})$.

We perform two steps of treatment to the raw data of vortex image shown in Fig. S2a in order to obtain a clear vortex image. Firstly, we subtracted the mapping of local conductance measured at zero voltage (Fig. S2(a)) by the one at 0.95 mV (Fig. S2(b)). After this process as shown in Fig. S2(c), the vortices look clearer than in the original image, but the influence of the bright chains along b-axis still remains. Secondly, we deducted the peaks caused by the bright chains in the Fourier transformation pattern from Fig. S2(c), then filled out the high frequency noise by performing a low-pass-filter, and finally inversed the Fourier transformation and got a clear image of vortices which are shown in Fig. 3(a).
